# Non-smoking adolescents’ perceptions of dissuasive cigarettes

**DOI:** 10.1016/j.abrep.2022.100433

**Published:** 2022-05-18

**Authors:** Dirk Jan A. van Mourik, Gera E. Nagelhout, Nikita L. Poole, Marc C. Willemsen, Math J.J.M. Candel, Crawford Moodie, Bas van den Putte, James F Thrasher, Hein de Vries

**Affiliations:** a Department of Health Promotion, Maastricht University (CAPHRI), Maastricht, The Netherlands; b IVO Research Institute, The Hague, The Netherlands; c Netherlands Expertise Centre for Tobacco Control (NET), Trimbos Institute, Utrecht, The Netherlands; d Department of Methodology and Statistics, Maastricht University (CAPHRI), Maastricht, The Netherlands; e Institute for Social Marketing and Health, University of Stirling, Stirling, UK; f Department of Communication, University of Amsterdam (ASCoR), Amsterdam, The Netherlands; g Department of Health Promotion, Education, and Behavior, Arnold School of Public Health, University of South Carolina, Columbia, USA

**Keywords:** Dissuasive cigarette, Health warning, Non-smoking adolescents

## Abstract

**Introduction:**

Dissuasive cigarettes, cigarettes with an unappealing colour or displaying a health warning label, may deter young people from smoking uptake.

**Methods:**

Two online surveys were conducted with non-smokers aged 12–17 to explore perceptions of cigarette appeal, harm and product trial. Study 1 was a within-subject study which examined perceptions of four cigarettes with different coloured paper, and four cigarettes displaying a warning. Study 2 was a between-subject study (with limited power), in which respondents were randomized to one of four cigarettes: (1) regular cigarette; (2) least favourable warning from Study 1; (3) least favourable colour from Study 1; or (4) a combination of the least favourable warning and colour from Study 1. Warnings or colours were considered least favourable when they had lower scores on appeal, harm, and product trial.

**Results:**

In Study 1, a cigarette featuring the warning ‘cancer, heart disease, stroke’ and a drab dark brown cigarette were rated lowest on appeal and trial intentions, and highest on perceived harm. In Study 2, there were no significant differences in perceptions of appeal, harm or trial intentions between the regular and dissuasive cigarettes.

**Conclusions:**

Findings from our within-subject study suggest that a cigarette displaying the text ‘cancer, heart disease, stroke’ and a drab dark brown coloured cigarette are most dissuasive for Dutch non-smoking adolescents. Whether dissuasive cigarettes reduce appeal, reduce product trial, or increase perceptions of harm compared to a regular cigarette should be further examined in larger between-subject studies.

## Highlights

• The marketing potential of cigarette packaging has been diminished by regulations.
• The cigarette stick can still be used to differentiate and promote tobacco products.
• Cigarette sticks can be designed to be dissuasive, e.g. making them drab dark brown.
• The text ‘cancer, heart disease, stroke’ on cigarettes was least appealing to youth.
• We could not demonstrate effects of dissuasive cigarettes among non-smoking youth.

## 1 Introduction

The marketing potential of cigarette packaging has, in many countries, been diminished by regulations, with large pictorial warnings common and an increasing number of countries requiring plain (or standardized) packaging (Canadian Cancer Society. Cigarette package health warnings. International Status Report). These measures have elevated the importance of the cigarette stick as a marketing tool for the tobacco industry. Features such as cigarette paper colour, decorative filter tips, and branding text and logos are used to promote products (Smith et al., 2017), and according to tobacco industry documents, are aimed at altering consumer perceptions of harm (Harris, 2011, Carpenter et al., 2005), appeal (Carpenter et al., 2005), pleasurableness (Bank, 1995), flavour and taste (Lewis and Wackowski, 2006). In response, and given the need for innovative developments in tobacco control (Beaglehole et al., 2015), researchers have begun to explore cigarettes that are designed to be ‘dissuasive’, for instance that are an unappealing colour or display a health warning label (Gallopel-Morvan et al., 2019). While marketing experts claim that dissuasive cigarettes may be a deterrent for youth and adolescents, unappealing for non-smokers and a signal that smoking is neither intelligent nor cool (Moodie, 2016), there remains a dearth of research on dissuasive cigarettes with adolescents.

Studies among adults have shown that textual warnings on cigarettes are perceived more effective than warnings on cigarette packages and are associated with increases in quitting intentions and reduced appeal of tobacco (Drovandi et al., 2019, Moodie et al., 2020, Drovandi et al., 2019, Hassan and Shiu, 2015). A relatively small number of studies on dissuasive cigarettes include adolescents under the age of 18, the minimum legal age for purchase in the Netherlands and many countries. Focus groups with female smokers from Scotland aged 16–24 years found that a cigarette displaying a warning was considered unappealing, a reminder of the health risks and off-putting given that it would be visible to others when smoking in public (Moodie et al., 2015). In the United Kingdom (UK), an in-home survey of smokers and non-smokers aged 11–16 years showed that most respondents believed that a cigarette displaying ‘smoking kills’ would deter people from smoking (Moodie et al., 2017). An online survey with smokers and non-smokers aged 16–24 years in the UK found that respondents perceived dissuasive cigarettes (green cigarette; cigarette displaying ‘smoking kills’) less favourably than a regular cigarette and reported being less likely to try one (Moodie et al., 2019). An online survey from Norway found that smokers and non-smokers aged 16–20 years perceived dissuasive cigarettes as less appealing, more harmful and worse tasting than a regular cigarette, and they indicated that they were less likely to try a dissuasive cigarette (Lund and Scheffels, 2018). An online survey with smokers and non-smokers aged 15–30 years from France revealed that, in comparison to branded cigarettes (regular, slim, pink), a grey cigarette was perceived as more harmful, less appealing, more likely to motivate smokers to quit or reduce smoking and less likely to motivate non-smokers to initiate with smoking (Gallopel-Morvan et al., 2019). However, none of the studies used realistic animations of cigarettes, or investigated whether the combination of a dissuasive colour and warning label would be more off-putting than a dissuasive colour or warning label alone. This study aims to fill these gaps by exploring perceptions of dissuasive cigarettes among a non-smoking adolescent population.

More is known about the impact of warning labels and colours on cigarette packages instead of on the cigarette itself. Systematic reviews on cigarette pack warning labels found that larger, more prominent, and pictorial warnings were associated with more knowledge of the harms of smoking, increased quitline calls and quit attempts, decreased smoking consumption, smoking prevalence, and smoking uptake (Noar et al., 2016, Hammond, 2011). One way to increase the salience of warning labels is via plain packaging. Systematic reviews showed that plain packaging increases the noticeability and effectiveness of the health warnings, as well as reduces the appeal of tobacco products, with some evidence that it also reduces misperceptions of harm as a consequence of pack design (Moodie et al., 2021, Moodie et al., 2012). Plain cigarette packs with a drab dark brown colour are perceived as the most unappealing and most harmful (Moodie et al., 2012).

In the current study, we focus on adolescents because this is when people are most inclined to try their first cigarette (Klein et al., 2013) with 67.2% of Dutch smokers initiating smoking between the ages of 12 and 16 years (Nuyts et al., 2018). Within this age group, 2.1% smoked daily, 7.8% smoked in the last month and 17.3% had ever smoked (Volksgezondheidenzorg.info, 2020). Early smoking uptake is associated with increased chances of stronger nicotine addiction in later life (Filippidis et al., 2015), lifetime smoking (Klein et al., 2013), and premature death (Thomson et al., 2020). Therefore, tobacco control innovations to prevent smoking uptake among adolescents are paramount (Beaglehole et al., 2015). A dissuasive cigarette may be a timely intervention to prevent smoking uptake as non-smoking adolescents will be exposed to this intervention when they are offered a cigarette. Accordingly, this study aims to examine perceptions of dissuasive cigarettes exclusively among non-smoking adolescents.

Our first objective was to describe which colour and warning label on cigarettes would be regarded as most dissuasive for non-smoking adolescents (Study 1). The second objective was to experimentally examine whether those exposed to dissuasive cigarettes were more likely to perceive such cigarettes as unattractive, harmful and less likely to be tried compared to those exposed to a regular cigarette (Study 2).

## 2 Methods

### 2.1 Sample

Respondents were non-smoking adolescents (not having smoked a cigarette at least once a month in the last six months). This was determined by asking whether the respondent had ever smoked a cigarette or part of it (yes; no). Those who answered positively were asked whether they smoked a cigarette at least once a month during the last six months (yes; no). Those who answered ‘yes’ to the last question were excluded from the study. Information about nationality (Dutch or other) was obtained. Those without Dutch nationality were excluded from the study, as we wanted to make sure all respondents could understand the Dutch language questionnaire.

Data collection was realised by Flycatcher, a commercial research agency. They collaborated with a partner agency (Panelclix) who provided additional respondents. Flycatcher has a quality mark for social research (quality standard ISO 20252) and Access Panels (ISO 26362). Flycatcher also uses the Integrity Code of the Expertise Center for Marketing Insights, Onderzoek (Research) and Analytics (MOA) and applies the Fair Data Privacy Code. This project received ethical approval from Maastricht University’s Faculty of Health Medicine and Lifesciences (FHML) – Research Ethics Committee (REC) (FHML-REC/2019/Mourik).

The parents or guardians of respondents were panel members from Flycatcher or Panelclix who were invited to participate via e-mail. After a parent or guardian completed the informed consent, they were asked to send the survey link to their child’s (thus the respondent’s) e-mail address. All respondents completed an informed consent prior to filling in the survey. The informed consent included information about the anonymous and voluntary nature of the study.

### 2.2 Study design

**Fig. 1:**
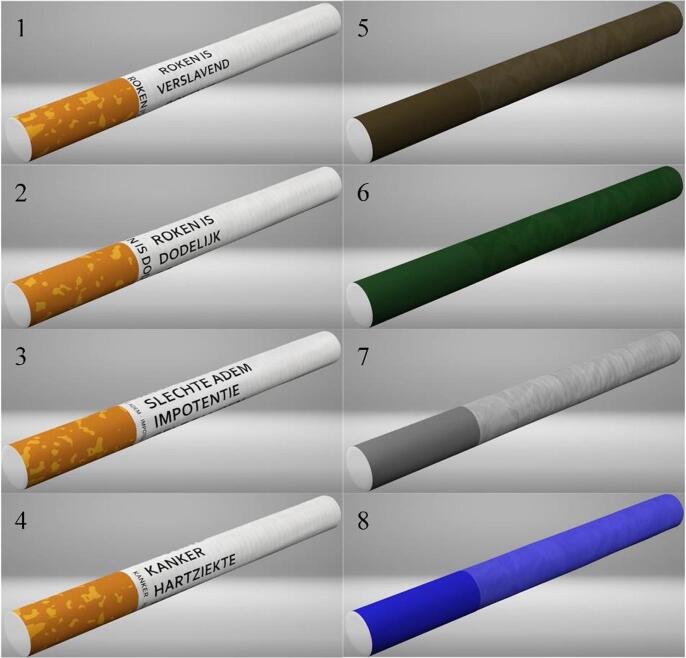
Cigarettes used in the first study, 1: smoking is addictive; 2: smoking kills; 3: impotence, yellow teeth, bad breath; 4: cancer, heart disease, stroke; 5: drab dark brown; 6: green; 7 grey; 8: blue. (For interpretation of the references to colour in this figure legend, the reader is referred to the web version of this article.)

Two separate online studies were conducted. Study 1 aimed to identify which colour and warning label on cigarettes would be the most dissuasive for non-smoking adolescents. Respondents were exposed to rotating, realistic animations of four cigarettes displaying different types of warning labels: 1) ‘smoking is addictive’, as addictiveness is an important attribute of the cigarette and research has shown that adolescents may have incorrect perceptions of the addictiveness of smoking (Wang et al., 2004, Rugkåsa et al., 2001); 2) ‘smoking kills’, because this is a concise and credible general message (Reitsma et al., 2017) used in previous studies (Hoek et al., 2016, Moodie et al., 2015, Moodie et al., 2017, Moodie et al., 2019, Lund and Scheffels, 2018); 3) ‘impotence, yellow teeth, bad breath’, because adolescents tend to respond to the short-term consequences of smoking (Farrelly et al., 2003); 4) ‘cancer, heart disease, stroke’, as these are among the most severe health risks of smoking (US Department of Health and Human Services (DHHS), 2014). The warning labels were displayed horizontally on the cigarette as a previous study suggested this orientation would have the greatest visibility (Moodie et al., 2015). The same warning label was also placed vertically above the filter to ensure the message remains intact once a cigarette is finished (Moodie et al., 2015). In Study 1, respondents were also shown cigarettes with different coloured papers. A drab dark brown colour was used as it is similar to the colour used for plain packaging (Moodie et al., 2012, Moon, 2011) and female smokers from New Zealand perceived similarly coloured cigarettes most unappealing in a qualitative study (Hoek et al., 2016, Hoek and Robertson, 2015). A green cigarette was used because this colour is associated with cigarettes being less appealing, lower quality, and more harmful (Moon, 2011), and in previous studies it has been rated less favourably than a regular cigarette and less likely to encourage product trial (Moodie et al., 2019, Lund and Scheffels, 2018). Third, a grey cigarette was used because this coloured has generally been found to be associated with greater harm (Gallopel-Morvan et al., 2019, Moon, 2011) and less appeal (Gallopel-Morvan et al., 2019, Moodie et al., 2012). Respondents were also exposed to a blue cigarette as this is not a natural colour and therefore may be dissuasive. Fig. 1 displays the cigarettes as shown in Study 1.

**Fig. 2:**
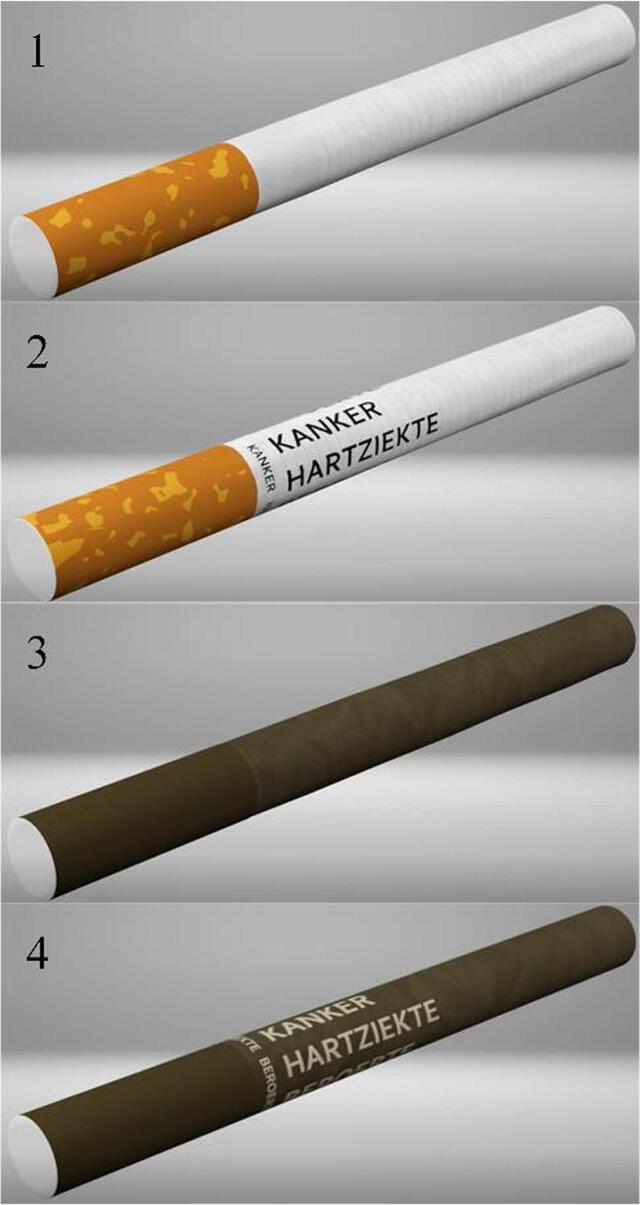
Cigarettes used in second study, 1: a regular cigarette; 2: cancer, heart disease, stroke; 3: drab dark brown; 4: drab dark brown combined with ‘cancer, heart disease, stroke’.

Study 2 used a between-subject design in which respondents were randomised to be exposed to one of four cigarettes: (1) regular; (2) least favourable warning label from Study 1; (3) least favourable colour from Study 1, or (4) a combination of the least favourable warning label and colour from Study 1 (Fig. 2). Warnings or colours were considered least favourable when they had lower scores on appeal, harm, and product trial.

### 2.3 Covariates

Information about gender (man or woman) and age (open question) were obtained. Educational attainment was assessed and categorised into primary education (1), practical education (2), preparatory secondary - vocational education (3), learning path support education (4), general secondary education (5), pre-university education (6), secondary vocational education (7), or other (8). Respondents were also asked ‘Does one of your parents or guardians smoke?’ (yes, every day; yes, some days; no; don’t know), which was dichotomised into ‘yes’ (first two answer categories) and ‘no’.

### 2.4 Measures regarding perceptions of dissuasiveness

#### 2.4.1 Appeal

Respondents were asked ‘How would you rate this cigarette?’. They could select one point on a seven-point semantic scale with anchors showing two extremes: 1) Unattractive (1) – Attractive (7); 2) Not stylish (1) – Stylish (7); 3) Not nice to be seen with (1) – Nice to be seen with (7); 4) Bad taste (1) – Nice taste (7); 5) Makes me feel bad (1) – Makes me feel good (7); 6) Would make me feel embarrassed (1) – Would not make me feel embarrassed (7) (Moodie et al., 2019). These items were summed into a composite score (Cronbach’s Alpha (α) in the first study = 0.89, α in the second study = 0.86), ranging from 6 (least appealing) to 42 (most appealing).

#### 2.4.2 Harm

To examine perceptions of harm, respondents were asked ‘How would you rate this cigarette?’. The seven-point semantic scale had anchors showing two extremes: 1) Does not look harmful to health (1) – Looks harmful to health (7); 2) Contains few harmful substances (1) – Contains many harmful substances (7); 3) Does not make me think about the dangers of smoking (1) – Makes me think about the dangers of smoking (7). These items were summed into a composite score (α in the first study = 0.80, α in the second study = 0.83), ranging from 3 (least harmful) to 21 (most harmful).

### 2.5 Interest in product trial

Respondents were asked ‘If a friend offered you this cigarette shown below, how likely would you be to try it?’, on a scale of 0 to 10 with anchors ‘No chance/almost no chance’ (0) and ‘Certain/practically certain’ (10) (Moodie et al., 2019, Juster, 1966). This is a relevant measure as most adolescents obtain cigarettes via their friends (van Dorsselaer et al., 2015), and this question is predictive of smoking initation among adolescents (Morello et al., 2018).

### 2.6 Procedure

In the first (within-subject) study, respondents were shown videos with animations of eight cigarettes (supplementary videos 1–8) and were asked about their perceptions. Respondents were first shown the animations of the cigarettes with warning labels (in the following order: ‘smoking is addictive’; ‘smoking kills’; ‘impotence, yellow teeth, bad breath’; ‘cancer, heart disease, stroke’) and thereafter the animations of the coloured cigarettes (in the following order: drab dark brown; green; grey; blue). The animations were shown on separate pages, with the possibility to go back to the previous page.

In the second (between-subject) study, respondents were randomised to be exposed to one of four animations of a cigarette (supplementary video 4, 5, 9, and 10) and asked about their perceptions of this cigarette. All questions had to be answered to complete the survey. At the end of both surveys, respondents were shown a video with information about why smoking is harmful (Clipphanger, 2017) and a link to a website with health information about smoking (Trimbos Institute, 2019). The first study was completed in April 2019, and the second in May 2019. By completing the survey, panel members (parent or guardian) received points they could redeem for a gift voucher. In addition, respondents were given a chance to win one of ten gift vouchers, each worth €17.50.

### 2.7 Analyses

Data were analysed with SPSS 23.0. For Study 1, mean scores were compared by using paired sample t-tests comparing the four warning texts with each other (6 comparisons for 3 outcomes = 18 comparisons) and comparing the four colours with each other (also 18 comparisons). To adjust for multiple comparisons (18 + 18 = 36 comparisons in total), pairwise differences were considered to be significant when the p-value was below 0.001 (=0.05/36). For the first study, 333 out of 1580 (21.1%) invitees completed the survey. Thirty-four respondents indicated smoking at least monthly during the last six months and two respondents were not Dutch. Those respondents were excluded from the study, leaving a sample of n = 297. A post-hoc power analysis shows that with this number of respondents there was more than 99% power to find a difference of at least 10% of the range of each outcome, which, for the observed correlations between outcomes, corresponded with minimum detectable effect sizes, that is, Cohen’s d_z_ (Lakens, 2013) of 0.41 for the colours and of 0.53 for the warning texts. The smallest difference that can be demonstrated with 297 respondents and 80% power was 5–6% of the range of each outcome measure, corresponding for these data to an effect size d_z_ of 0.24–0.25, which can be considered small (Cohen, 1988). We can conclude that study 1 had sufficient power to detect even small effect sizes.

For study 1, we also performed secondary analyses to examine whether, on average, texts scored differently on appeal, harm, and product trial as compared to colours. For these secondary analyses, we pooled scores across all four colours and across all four warning texts and performed paired sample t-tests on these averaged scores for each outcome. To adjust for multiple comparisons (3 outcomes), differences were considered to be significant when the p-value was below 0.017 (=0.05/3).

For Study 2, analysis of covariance (ANCOVA’s) was performed with cigarette design as the independent variable, perceptions as dependent variable, and gender, age, educational attainment, ever tried smoking and having a parent or guardian who smokes as covariates. To adjust for multiple comparisons (3 outcomes), estimates were considered to be significant when the p-value was below 0.017 (=0.05/3). For the second study, 316 out of 1363 (23.2%) invitees answered the survey. Thirty-five respondents indicated smoking at least monthly during the last six months, and one respondent was not Dutch. Those respondents were excluded from the study, leaving a sample of n = 280. A post-hoc power analysis shows that with this number of respondents there was 31–33% power to find a difference of at least 10% of the range of each outcome measure between at least two groups, which for the R^2^ values of the regression of the outcomes on the covariates of the ANCOVA, corresponded with an effect size f (Cohen, 1988) of 0.14 for all outcome measures, which can be considered small. The smallest difference between at least two groups that can be demonstrated with 280 respondents and 80% power was 16–17% of the range of each outcome measure, corresponding for these to an effect size f of 0.22, which can be considered medium (Cohen, 1988). Concluding, for small effect sizes study 2 had insufficient power, and thus the results of this study should be considered indicative.

## 3 Results

Supplementary table 1 provides an indication that respondents were representative of the Dutch population aged 12–17 years. Table 1 shows the sample characteristics of both studies. The mean age was 14.6 years for respondents in Study 1 and 15.1 years for respondents in Study 2. The highest percentage of respondents were enrolled in preparatory secondary vocational education (25.9% in Study 1 and 28.6% in Study 2). Almost all respondents indicated that they had never smoked (92.9% in Study 1 and 90.7% in Study 2).

**Table 1:** Sample characteristics.

	**1st study**	**2nd study**
**Gender**		
Female	140 (47.1%)	141 (50.4%)
Male	157 (52.9%)	139 (49.6%)
**Age**		
12	45 (15.2%)	21 (7.5%)
13	50 (16.8%)	39 (13.9%)
14	44 (14.8%)	41 (14.6%)
15	51 (17.2%)	45 (16.1%)
16	57 (19.2%)	66 (23.6%)
17	50 (16.8%)	68 (24.3%)
**Education**		
Primary education	27 (9.1%)	15 (5.4%)
Practical education	7 (2.4%)	10 (3.6%)
Preparatory secondary - vocational education	77 (25.9%)	80 (28.6%)
Learning path support education	6 (2.0%)	3 (1.1%)
General secondary education	66 (22.2%)	66 (23.6%)
Pre-university education	74 (24.9%)	71 (25.4%)
Secondary vocational education	33 (11.1%)	27 (9.6%)
Other	7 (2.4%)	8 (2.9%)
**Ever smoked**		
Yes	21 (7.1%)	26 (9.3%)
No	276 (92.9%)	254 (90.7%)
**Smoking parent**		
Yes	80 (26.9%)	91 (32.5%)
No	217 (73.1%)	188 (67.1%)
Don’t know	0 (0%)	1 (0.4%)

**Study 1.**

Table 2 shows that regarding the cigarettes with warning labels, the cigarette displaying ‘cancer, heart disease, stroke’ scored lowest on appeal (mean = 8.57, standard deviation (SD) = 4.81), and interest in product trial (mean = 1.64, SD = 1.53), and highest on harm (mean = 17.62, SD = 5.34). These differences were significant for all three indicators (appeal, product trial, and harm) when comparing this warning with the warnings ‘smoking is addictive’ and ‘smoking kills’. Differences for appeal and product trial were not significant when comparing the cigarettes with the text ‘cancer, heart disease, stroke’ with ‘impotence, yellow teeth, bad breath’; both these cigarettes were considered very unappealing, and few respondents were interested in product trial. Regarding colour, Table 3 shows that the drab dark brown cigarette scored lowest on appeal (mean = 10.80, SD = 7.31) and interest in product trial (mean = 1.78, SD = 1.70), and the highest on harm (mean = 16.14, SD = 5.49). These differences were significant for all three indicators (appeal, product trial, and harm) when comparing the drab dark brown cigarette with the grey and blue cigarette. Differences for appeal and product trial were not significant when comparing the drab dark brown cigarette with the green cigarette; both these cigarettes were considered very unappealing, and few respondents were interested in product trial.

**Table 2:** Means, standard deviations, and results from paired t-tests for the cigarettes with different warning texts in Study 1.

	‘Smoking is addictive’ (1) Mean (SD)	‘Smoking kills’ (2) Mean (SD)	‘Impotence, yellow teeth, bad breath’ (3)Mean (SD)	‘Cancer, heart disease, stroke’ (4)Mean (SD)	1 vs 2	1 vs 3	1 vs 4	2 vs 3	2 vs 4	3 vs 4
**Appeal (ranging from 6 to 42)**	11.28 (6.37)	9.82 (5.83)	9.02 (5.12)	8.57 (4.81)	t = 7.136, p < 0.001	t = 9.148, p < 0.001	t = 10.048, p < 0.001	t = 5.000, p < 0.001	t = 5.884, p < 0.001	t = 3.093, p = 0.002
**Harm (ranging from 3 to 21)**	15.67 (5.40)	16.80 (5.40)	17.05 (5.13)	17.62 (5.34)	t = -5.644, p < 0.001	t = -6.307, p < 0.001	t = -7.621, p < 0.001	t = -1.600, p = 0.111	t = -4.166, p < 0.001	t = -3.631, p < 0.001
**Product trial (ranging from 0 to 10)**	1.97 (1.66)	1.91 (1.77)	1.77 (1.53)	1.64 (1.53)	t = 0.943, p = 0.347	t = 3.059, p = 0.002	t = 4.025, p < 0.001	t = 2.862, p = 0.005	t = 4.180, p < 0.001	t = 2.903, p = 0.004

**Table 3:** Means, standard deviations, and results from paired t-tests for the cigarettes with different colours in Study 1.

	Drab dark brown (5)Mean (SD)	Green (6) Mean (SD)	Grey (7) Mean (SD)	Blue (8) Mean (SD)	5 vs 6	5 vs 7	5 vs 8	6 vs 7	6 vs 8	7 vs 8
**Appeal (ranging from 6 to 42)**	10.80 (7.31)	11.83 (7.66)	13.06 (8.44)	14.01 (9.05)	t = -3.290, p = 0.001	t = -6.028, p < 0.001	t = -6.727, p < 0.001	t = -3.305, p = 0.001	t = -5.481, p < 0.001	t = -2.183, p = 0.030
**Harm (ranging from 3 to 21)**	16.14 (5.49)	15.33 (5.60)	15.32 (5.42)	14.81 (5.72)	t = 4.482, p < 0.001	t = 4.087, p < 0.001	t = 5.182, p < 0.001	t = 0.066, p = 0.948	t = 2.578, p = 0.010	t = 2.326, p = 0.021
**Product trial (ranging from 0 to 10)**	1.78 (1.70)	1.87 (1.72)	2.00 (1.86)	2.09 (1.99)	t = -2.064, p = 0.040	t = -4.042, p < 0.001	t = -4.168, p < 0.001	t = -2.754, p = 0.006	t = -3.538, p < 0.001	t = -1.310, p = 0.191

Secondary analyses of study 1, in which we averaged scores across all four colours and across all four warning texts showed significant differences between cigarettes with colours versus cigarettes with warning texts on appeal (t = -8.45, p < 0.001) and harm (t = 5.98, p < 0.001), but not on product trial (t = -2.05, p = 0.041). The coloured cigarettes were perceived as more appealing (mean = 12.43, SD = 6.97) than the cigarettes with warning texts (mean = 9.67, SD = 5.10), and were also perceived as less harmful (mean = 15.40, SD = 5.09) than the cigarettes with warning texts (mean = 16.78, SD = 4.88).

**Study 2.**

The four randomised groups did not differ based on gender, age, education, having ever smoked, and having a parent or guardian who smokes (Supplementary table 2). Table 4 shows the means and standard deviations of perceptions of the four cigarettes. Based on the basis on Study 1, the four cigarettes in Study 2 were: (1) regular cigarette; (2) drab dark brown cigarette; (3) cigarette with warning ‘cancer, heart disease and stroke’, or (4) a drab dark brown cigarette with the warning ‘cancer, heart disease and stroke’. ANCOVA’s revealed no differences between the cigarettes for the composite scores on appeal (F = 0.387, p = 0.762) harm (F = 0.774, p = 0.510), or interest in product trial (F = 0.473, p = 0.701).

**Table 4:** Means, standard deviations, and results from ANCOVA’s for the four cigarettes in Study 2.

	Regular cigarette (n = 69) Mean (SD)	‘Cancer, heart disease, stroke’ (n = 69) Mean (SD)	Drab dark brown cigarette (n = 65) Mean (SD)	Drab dark brown cigarette combined with ‘cancer, heart disease, stroke’ (n = 77)Mean (SD)	ANCOVA results
**Appeal (ranging from 6 to 42)**	11.15 (6.45)	10.94 (6.06)	11.72 (6.56)	11.61 (7.08)	F = 0.387, p = 0.762
**Harm (ranging from 3 to 21)**	14.67 (5.85)	15.63 (5.51)	14.52 (6.22)	15.60 (5.66)	F = 0.774, p = 0.510
**Product trial (ranging from 0 to 10)**	1.99 (1.58)	2.30 (2.19)	2.02 (1.74)	1.88 (2.02)	F = 0.473, p = 0.701

## 4 Discussion

In the first (within-subject) study we found that drab dark brown was perceived as a more dissuasive colour for cigarettes than green, grey or blue (ordered from most to least dissuasive). This finding is in line with research with adult smokers in New Zealand, who perceived drab dark brown cigarettes the most unappealing (Hoek et al., 2016, Hoek and Robertson, 2015), a survey on cigarette packaging with Australian adult smokers (Moon, 2011), and a systematic review on plain packaging which showed that cigarette packs this colour were perceived as the most unappealing and most harmful (Moodie et al., 2012). We also found that a cigarette displaying the text ‘cancer, heart disease, stroke’ was perceived as more dissuasive among our sample than cigarettes displaying warnings about smoking causing impotence, yellow teeth and bad breath, or smoking being deadly or addictive (ordered from most to least dissuasive). Additionally, secondary analyses showed that cigarettes with warning texts were perceived as less appealing and more harmful than cigarettes with colours.

We found no significant differences in the second study between regular and dissuasive cigarettes, unlike previous research that did suggest that dissuasive cigarettes may have the potential to deter smokers (Hoek et al., 2016, Moodie et al., 2017, Moodie et al., 2019, Lund and Scheffels, 2018) and non-smokers (Moodie et al., 2017, Moodie et al., 2019, Lund and Scheffels, 2018). Research design and a lack of power may offer an explanation. In within-subject studies, as is implemented in previous studies (Moodie et al., 2019, Lund and Scheffels, 2018, Borland and Savvas, 2013), respondents are able to compare dissuasive cigarettes with a regular cigarette, which may influence their perceptions, whereas respondents in our (second) between-subject study were exposed to only one cigarette and therefore unable to do so. We think that a between-subject design is most suitable for future studies, as dissuasive cigarettes are a potential future policy measure that would be implemented at a national or even supranational (e.g. European) level. Inhabitants would then be exposed to only one type of dissuasive cigarette unless they travel to another country without this policy.

With respect to the sample, more than 90% were never-smokers. Never smokers may have a more negative attitude towards smoking than ever-smokers, such that there is very little interest in trial of any cigarette. This is reflected in the low scores found in this study, where all cigarettes are perceived as extremely unappealing and harmful, and there could thus be a floor effect. Consistent with this, a longitudinal study of Dutch adolescents found that past smoking moderately affected attitudes in the direction of holding less negative attitudes towards smoking (De Leeuw et al., 2008). In addition, a systematic review reports that non-smokers typically find cigarettes to be less appealing than occasional smokers (Drovandi et al., 2018). The small sample size for the second study, where approximately 70 participants were exposed to each cigarette, also meant that there was insufficient power to detect smaller differences between cigarettes.

Strengths of our study include the use of rotating, realistic animations, which may have been conducive to imagining how the cigarette would look like in real life, and the use of an experimental design, which provided internally-valid evidence. There are also limitations that should be taken into account when interpreting the results. First, our findings are not generalizable to other populations, such as current smokers or other cultures or age groups. Additionally, participation rates were low and so this may have caused underrepresentation in the sample in other ways. Second, respondents were not able to handle the cigarettes, which would have allowed them to have a true representation of dimension, colour, and tactility (Ford et al., 2014). Third, the sample size in Study 2 is small, with <70 participants exposed to three of the cigarettes, resulting in insufficient power. Fourth, the novelty of the stimuli, and forced exposure, may have influenced responses. Fifth, there was no randomisation of the order of the presentation of stimuli in Study 1, which may have influenced the results. Sixth, we have not systematically combined colours and warning texts in the stimuli and can thus not conclude anything about possible interactions between colours and texts. Finally, respondents were enrolled through their parents or guardians who were part of a panel and who received points -that they could redeem for a gift voucher- when their child participated in the survey. Although we explained the voluntary nature of participation, this may have caused some pressure to participate.

Future research could build upon this study by exploring the impact of dissuasive cigarettes among larger samples of susceptible and non-susceptible never smokers. It is also important to conduct research on dissuasive cigarettes in parts of the world where single cigarettes are sold, for instance in markets such as India (Lal et al., 2015) and much of Africa (Wherry et al., 2014, African Tobacco Control Alliance, 2018). In such markets, dissuasive cigarettes might be even more important as a deterrent and possible source of information about the health risks of smoking because people are not necessarily exposed to packs. In addition, research is important in different parts of the world given that colour preferences differ between cultures (Aslam, 2006). Future research could also compare dissuasive cigarettes with regular cigarettes and other types of cigarettes that may increase appeal among younger people (Moodie et al., 2019), such as slimmer cigarettes or cigarettes with flavour-changing capsules in the filters.

Findings from our within-subject study suggest that a cigarette displaying the text ‘cancer, heart disease, stroke’ and a drab dark brown coloured cigarette are most dissuasive for Dutch non-smoking adolescents. However, our between-subject study did not reveal any significant differences in perceptions between a regular cigarette and dissuasive cigarettes, which may have been due to a lack of power in this second study. Future studies should further examine the potential effectiveness of dissuasive cigarettes among other populations, and with larger samples.

## 5 Human subjects approval statement

This project received ethical approval from Maastricht University’s Faculty of Health Medicine and Lifesciences (FHML) – Research Ethics Committee (REC) (FHML-REC/2019/Mourik). The studies described within this manuscript meet the ethical standard outlines in Helsinki Declaration of 1975 as revised in 2000. Both the parent/guardian of the respondent and the respondent gave informed consent.

### CRediT authorship contribution statement

**Dirk Jan A. van Mourik:** Conceptualization, Methodology, Formal analysis, Investigation, Writing – original draft, Funding acquisition. **Gera E. Nagelhout:** Conceptualization, Methodology, Writing – review & editing, Supervision. **Nikita L. Poole:** Writing – review & editing. **Marc C. Willemsen:** Methodology, Writing – review & editing, Supervision. **Math J. J. M. Candel:** Methodology, Formal analysis, Writing – review & editing. **Crawford Moodie:** Methodology, Writing – review & editing. **Bas van den Putte:** Methodology, Writing – review & editing. **James F Thrasher:** Methodology, Writing – review & editing. **Hein de Vries:** Methodology, Writing – review & editing, Supervision.

## Declaration of Competing Interest

The authors declare that they have no known competing financial interests or personal relationships that could have appeared to influence the work reported in this paper.
