# Supplementary material for: Non-smoking adolescents’ perceptions of dissuasive cigarettes
Source: Addict Behav Rep. 2022 May 18;15:100433. doi: 10.1016/j.abrep.2022.100433 (PMC9127256; doi:10.1016/j.abrep.2022.100433)
Supplement: Supplementary data 11 [file mmc11.docx]

Supplementary video 1: <https://youtu.be/cKxArsKALfU>

Supplementary video 2: <https://youtu.be/LqpFZpWgpjI>

Supplementary video 3: <https://youtu.be/1hgm8fMDEWk>

Supplementary video 4: <https://youtu.be/8hP7fLCsQfA>

Supplementary video 5: <https://youtu.be/33R1t5X6jck>

Supplementary video 6: <https://youtu.be/PPz9QwgQuKc>

Supplementary video 7: <https://youtu.be/XD0OS2YRUYE>

Supplementary video 8: <https://youtu.be/tP2KQEvrosk>

Supplementary video 9: <https://youtu.be/v3GnYqnhGJY>

Supplementary video 10: <https://youtu.be/SFsjwYgW8nQ>
