# Supplementary material for: Non-smoking adolescents’ perceptions of dissuasive cigarettes
Source: Addict Behav Rep. 2022 May 18;15:100433. doi: 10.1016/j.abrep.2022.100433 (PMC9127256; doi:10.1016/j.abrep.2022.100433)
Supplement: Supplementary data 12 [file mmc12.docx]

**Supplementary table 1.** Sample characteristics of respondents from the first and second study compared with data from the CBS. *

|  | **Study 1** | **Study 2** | **CBS** |
| --- | --- | --- | --- |
| **Gender** |  |  |  |
| Female | 47% | 50% | 51% |
| Male | 53% | 50% | 49% |
| **Age** |  |  |  |
| 12 | 15% | 8% | 16% |
| 13 | 17% | 14% | 17% |
| 14 | 15% | 15% | 17% |
| 15 | 17% | 16% | 17% |
| 16 | 19% | 24% | 17% |
| 17 | 17% | 24% | 17% |
| **Province** |  |  |  |
| Drenthe | 3% | 4% | 3% |
| Flevoland | 6% | 1% | 3% |
| Friesland | 6% | 5% | 4% |
| Gelderland | 11% | 8% | 13% |
| Groningen | 4% | 5% | 3% |
| Limburg | 11% | 13% | 6% |
| Noord – Brabant | 15% | 16% | 15% |
| Noord – Holland | 9% | 15% | 16% |
| Overijssel | 8% | 8% | 7% |
| Utrecht | 6% | 6% | 8% |
| Zeeland | 3% | 3% | 2% |
| Zuid – Holland | 18% | 19% | 21% |

* CBS = Statistics Netherlands. The figures used to determine representativeness are retrieved from the so-called "Golden Standard". This is a calibration tool specially developed by the Center for Marketing Insights, Research & Analytics (MOA) in collaboration with Statistics Netherlands.
