# Supplementary material for: Non-smoking adolescents’ perceptions of dissuasive cigarettes
Source: Addict Behav Rep. 2022 May 18;15:100433. doi: 10.1016/j.abrep.2022.100433 (PMC9127256; doi:10.1016/j.abrep.2022.100433)
Supplement: Supplementary data 13 [file mmc13.docx]

**Supplementary table 2**. Sample characteristics of the four randomised groups from the second study. *

|  | **1** (N = 69) | **2** (N = 69) | **3** (N = 65) | **4** (N = 77) |  |
| --- | --- | --- | --- | --- | --- |
| **Gender** |  |  |  |  |  |
| Female | 32 (46.4%) | 32 (46.4%) | 35 (53.8%) | 42 (54.5%) | χ2 = 1.731 |
| Male | 37 (53.6%) | 37 (53.6%) | 30 (46.2%) | 35 (45.5%) | p = 0.630 |
| **Age** (mean) |  |  |  |  | F = 2.126 |
|  | 15.41 (1.57) | 15.07 (1.65) | 14.71 (1.55) | 15.08 (1.63) | p = 0.097 |
| **Education** |  |  |  |  |  |
| Primary education | 2 (2.9%) | 5 (7.2%) | 2 (3.1%) | 6 (7.8%) | χ2 = 20.902 |
| Practical education | 3 (4.3%) | 2 (2.9%) | 2 (3.1%) | 3 (3.9%) | p = 0.465 |
| Preparatory secondary -  vocational education | 16 (23.2%) | 23 (33.3%) | 17 (26.2%) | 24 (31.2%) |  |
| Learningpath support  education | 1 (1.4%) | 0 (0%) | 1 (1.5%) | 1 (1.3%) |  |
| General secondary education | 20 (29.0%) | 12 (17.4%) | 13 (20.0%) | 21 (27.3%) |  |
| Pre-university education | 18 (26.1%) | 16 (23.2%) | 24 (36.9%) | 13 (16.9%) |  |
| Secondary vocational education | 8 (11.6%) | 6 (8.7%) | 5 (7.7%) | 8 (10.4%) |  |
| Other | 1 (1.4%) | 5 (7.2%) | 1 (1.5%) | 1 (1.3%) |  |
| **Ever smoked** |  |  |  |  |  |
| Yes | 6 (8.7%) | 9 (13.0%) | 7 (10.6%) | 4 (5.2%) | χ2 = 2.885 |
| No | 63 (91.3%) | 60 (87.0% | 58 (89.2%) | 73 (94.8%) | p = 0.410 |
| **Smoking parent or guardian** |  |  |  |  |  |
| Yes | 19 (27.5%) | 21 (30.4%) | 24 (36.9%) | 27 (35.1%) | χ2 = 4.735 |
| No | 50 (72.5%) | 47 (68.1%) | 41 (63.1%) | 50 (64.9%) | p = 0.578 |
| Don’t know | 0 (0%) | 1 (1.4%) | 0 (0%) | 0 (0%) |  |

*1: a regular cigarette; 2: cancer, heart disease, stroke; 3: drab dark brown; 4: drab dark brown combined with ‘cancer, heart disease, stroke’.
